# Development of the Tele-Measurement of Plasma Uniformity via Surface Wave Information (TUSI) Probe for Non-Invasive In-Situ Monitoring of Electron Density Uniformity in Plasma Display Fabrication Process

**DOI:** 10.3390/s23052521

**Published:** 2023-02-24

**Authors:** Si-Jun Kim, Min-Su Choi, Sang-Ho Lee, Won-Nyoung Jeong, Young-Seok Lee, In-Ho Seong, Chul-Hee Cho, Dae-Woong Kim, Shin-Jae You

**Affiliations:** 1 Applied Physics Lab for PLasma Engineering (APPLE), Department of Physics, Chungnam National University, Daejeon 34134, Republic of Korea; 2 Department of Plasma Engineering, Korea Institute of Machinery and Materials (KIMM), Daejeon 34104, Republic of Korea; 3 Institute of Quantum Systems (IQS), Chungnam National University, Daejeon 34134, Republic of Korea

**Keywords:** plasma diagnostics, electron density, electron density uniformity, plasma uniformity, planar microwave probe, in-situ plasma process monitoring

## Abstract

The importance of monitoring the electron density uniformity of plasma has attracted significant attention in material processing, with the goal of improving production yield. This paper presents a non-invasive microwave probe for in-situ monitoring electron density uniformity, called the Tele-measurement of plasma Uniformity via Surface wave Information (TUSI) probe. The TUSI probe consists of eight non-invasive antennae and each antenna estimates electron density above the antenna by measuring the surface wave resonance frequency in a reflection microwave frequency spectrum (S${}_{11}$). The estimated densities provide electron density uniformity. For demonstration, we compared it with the precise microwave probe and results revealed that the TUSI probe can monitor plasma uniformity. Furthermore, we demonstrated the operation of the TUSI probe beneath a quartz or wafer. In conclusion, the demonstration results indicated that the TUSI probe can be used as an instrument for a non-invasive in-situ method for measuring electron density uniformity.

## 1. Introduction

Plasma technologies in the modern industry have been applied in tremendous fields such as material fabrication, medical treatment, nuclear fusion energy, and environment pollution controls [1,2]. Among them, plasma processing has played a significant role in the state-of-the-art semiconductor fabrication, for instance, high-aspect-ratio contact hole etching [3,4,5,6]. Recently, plasma process monitoring has become significant since the loss of a production yield induced by anomalous process behaviors is no longer negligible, such as arcing, hunting, and process drift [7,8,9,10,11]. To improve the process yield, advanced process control (APC) based on real-time process monitoring devices has been adopted and is being developed [12,13,14]. To minimize process perturbation, non-invasive monitoring parameters have been utilized in the APC technology, such as voltages applied and currents flowing through the discharge electrode or antenna, capacitor positions in an impedance matcher, optical emission spectra, throttle valve positions, gas flow rates, and plasma parameters, such as electron density and temperature. Tremendous monitoring data are gathered and processed to monitor *process stability* [12,15,16,17,18,19].

Recently, process stability management *over wafer level* has been required to further improve the production yield. Thus, process uniformity monitoring becomes significant. Among those monitoring parameters, electron density—which is directly related to processing time and quality—is a significant factor [13,16,17,20]. Thus, electron density uniformity is a critical factor for process uniformity [21]. Several non-invasive techniques for measuring electron density uniformity have been developed. For instance, Mahoney et al. developed a non-invasive planar double Langmuir probe array for measuring electron density on the wafer level [22]. It measures electron current from plasma and derive electron density from the current. Thus, this probe is not applicable for in-situ plasma process monitoring due to signal reduction by contaminant deposition on the probe surface. Kim et al. [23] developed a 2-dimensional non-invasive probe array, the measurement principle of which is based on the floating harmonic method. A sinusoidal voltage wave with the frequency $\omega_{1}$ is applied to a probe and harmonic currents containing $\omega_{1}$, 2$\omega_{1}$, and higher harmonics are recorded to derive electron density. Each probe’s harmonic current data provides the uniformity of plasma parameters. As this method uses several kilohertz voltage waves, it can work under the contaminant deposition environment. It is, however, vulnerable to radio-frequency (RF) noise from the discharge electrode or antenna because it uses a lower frequency than the RF. In addition to an electrical probe, Kim et al. [24] proposed an optical method with a revolving module, which is a rotational slit for blocking the viewport area of a process chamber. By rotating the revolving slit, plasma emission section by section can be recorded, thereby estimating plasma intensity uniformity. Despite its simple installment, intensity monitoring is not suitable for precise electron density measurement since plasma emission depends on electron density, gas density, and temperature. Although they can estimate plasma uniformity, they are not suitable for the in-situ plasma process monitoring.

A microwave probe is a good candidate for an in-situ monitoring method applicable under a contaminant deposition and RF noise environments [16,25]. Several non-invasive microwave probes have been developed, such as the planar cutoff probe [20], the curling probe [26], and the planar multipole resonance probe [27]; they represented a good performance for in-situ measurement of the electron density. However, an array-type microwave probe has yet to be developed due to its complicated design and large antenna size. Recently, Kim et al. have developed the measurement of lateral electron density (MOLE) probe, which has a simple probe design and small antenna size [12]. Adopting the MOLE probe’s advantages, this paper proposes a non-invasive in-situ microwave probe that can measure plasma uniformity, called the Telemeasurement of plasma Uniformity via Surface wave Information (TUSI) probe.

In the following section, we will provide a detailed examination of the configuration and operating principles of the TUSI probe. The third section will present an overview of the experimental setup used for demonstration, as well as a comparison of the results obtained with the TUSI probe against those obtained with a precise microwave probe. In the fourth section, we will demonstrate the in-situ capabilities of the TUSI probe by analyzing results obtained with a quartz and a wafer placed on the probe. Finally, in the last section, we will summarize the findings of this study and present our conclusions.

## 2. Configuration

**Figure 1:**
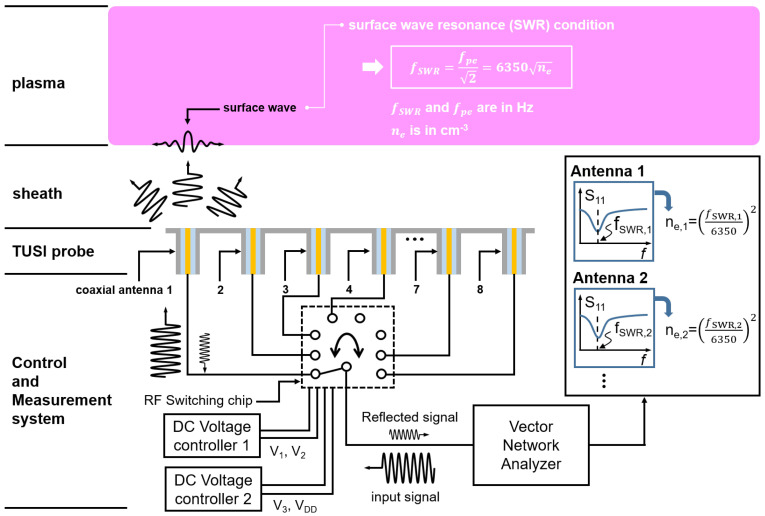
Schematic diagram for the concept of the TUSI probe.

Figure 1 shows a schematic diagram for the TUSI probe. The TUSI probe has eight coaxial antennae connected to the control and measurement system. This system consists of the RF switch chip (SKY13418-485LF Skyworks Solutions, Irvine, California, United States), two direct current (DC) voltage controllers having two voltage outputs (ED-200E, ED Laboratory, unknown), and a vector network analyzer (E5071B, Agilent Inc., Santa Clara, CA, United States). Here, using the RF switch chip, even a single vector network analyzer can drive eight antennas. For the switching chip operation, four direct current (DC) voltages are applied; three (V${}_{1}$, V${}_{2}$, and V${}_{3}$) are for port switching and the last one (V${}_{\mathrm{DD}}$) the chip power supply. The control logic is well organized in Table 1. For instance, V${}_{1}$, V${}_{2}$, and V${}_{3}$ outputs 0 V, 1.8 V, and 1.8 V, respectively, for switching to the 4th antenna. Furthermore, the switching chip has nine input/output (I/O) ports; one is connected to the vector network analyzer and the others to eight coaxial antennae.

The measurement principle of the TUSI probe is shared with the MOLE probe—the surface wave resonance frequency measurement [12,28]—and its operation is as follows. In the case of switching to the antenna 1, the vector network analyzer sends the input sinusoidal signal with frequency, *f*, and it radiates inside the sheath as shown in Figure 1. Here, the sheath is the ion-space charge region between the plasma and material surface facing plasma, for instance, the TUSI probe. A part of the radiated wave converts to the surface wave at the sheath–plasma interface when the *f* is lower than the surface wave resonance (SWR) frequency ($f_{\mathrm{SWR}}$). Here, the $f_{\mathrm{SWR}}$ is defined as

(1) $$f_{\mathrm{SWR}}=\frac{f_{\mathrm{pe}}}{\sqrt{2}},$$

where the $f_{\mathrm{pe}}$ is the electron plasma oscillation frequency defined as

(2) $$f_{\mathrm{pe}}=8980\sqrt{n_{e}},$$

where $n_{e}$ is the electron density per cubic centimeter [3]. The vector network analyzer sweeps the *f* and simultaneously measures the reflection microwave frequency spectrum (S${}_{11}$) over the *f*. The measured S${}_{11}$ has the dip, as shown in Figure 1, due to the strong electromagnetic wave confinement on the interface and absorption by the plasma at the SWR condition [12]. Thus, the electron density above the antenna 1 ($n_{e,1}$) can be estimated by using Equation (1). Then, the DC voltage controllers switch to the second antenna and the electron density above the antenna 2 ($n_{e,2}$) can be estimated with the same procedure. As a result, the whole estimation from the first and eighth antenna provides electron density uniformity.

**Figure 2:**
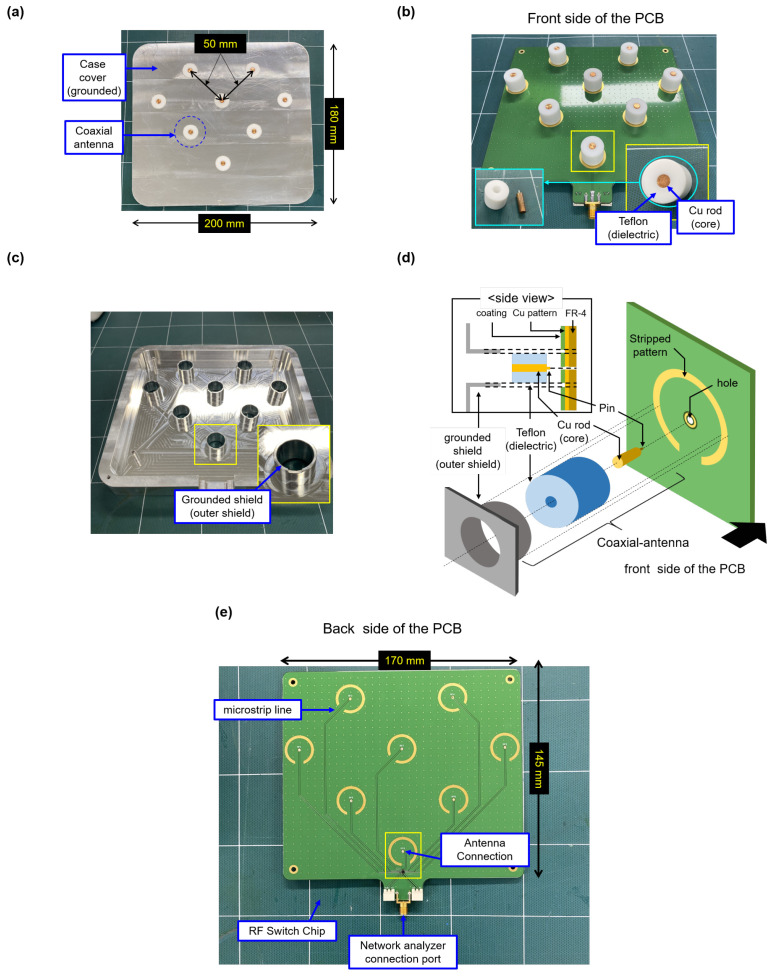
Photographs of (**a**) appearance of the TUSI probe, (**b**) coaxial antenna parts and the front side of the printed circuit board (PCB), and (**c**) the backside of the case cover. (**d**) Schematic diagram of the components of the coaxial antenna (the Cu rod(core), the Teflon (dielectric), and the grounded shield (outer shield)) and its installment to the PCB. (**e**) Top view of the back-side of the PCB.

Figure 2 represents the photographs of the TUSI probe and its parts. Figure 2a shows the top view of the appearance of the TUSI probe. The TUSI probe has the outer size of 180 × 200 mm${}^{2}$ and inter-diagonal distances between antennae are 50 mm. As its top surface is flat, the TUSI probe is a non-invasive method. Figure 2b shows the printed circuit board (PCB) and parts of the coaxial antenna composed of the Cu rod (core) and the Teflon (dielectric). The inside of the case cover has hollow cylinders, playing the role of the grounded shield of the coaxial antenna, as shown in Figure 2c,d. We mounted the pin of the Cu rod to the hole with the soldering (Figure 2d). Furthermore, the stripped pattern improves the electric connection of the hollow cylinder to the ground of the PCB. Here, there are stripped patterns in both sides. In fact, they are fabricated for simply examining which PCB side is the best for the RF noise reduction. As it is not the main point of this paper, we simply discussed it as follows. Based on the simple test, the current setup (front side is up and back side down) shows huge RF noise reduction. This results from the main signal lines being able to be shielded by the grounded plates in the front side of the PCB; except for the holes on the front PCB, the resting area is a grounded plate.

In this system, it is the key point that dominant wave reflection only occurs at the end of the antenna. This means that the characteristic impedances of all parts have to be 50 Ω, called impedance matching. For the matching, a freeware, QucsStudio [29], was adopted for calculating the geometric parameters of a micro-strip line; in this case, a coplanar waveguide with the backside; a pattern thickness of 70 μm, the central line width of 0.7 mm, and distance of central to ground lines of 0.2 mm. Here, the PCB height is 1.6 mm and the substrate material is FR-4 with a relative dielectric constant of 4.5. Furthermore, the coaxial antenna geometry parameters were as follows: the relative dielectric constant of the Teflon was 2.1 and the outer diameters of the Cu rod and the Teflon were 5.0 mm and 16.7 mm, respectively.

In general, S${}_{11}$ decreases over frequency [12] since a coaxial cable has the internal loss by absorption. Thus, the calibration of the coaxial cable connecting the PCB and the VNA is significant for achieving a high signal-to-noise ratio. Specifically, before mounting the coaxial cable to the network analyzer connection port shown in Figure 2e, calibration at the end of the cable enables us to remove the internal loss effect and initialize the S${}_{11}$ spectrum.

## 3. Validation of the TUSI Probe with the Cutoff Probe

### 3.1. Experiment Setup

**Figure 3:**
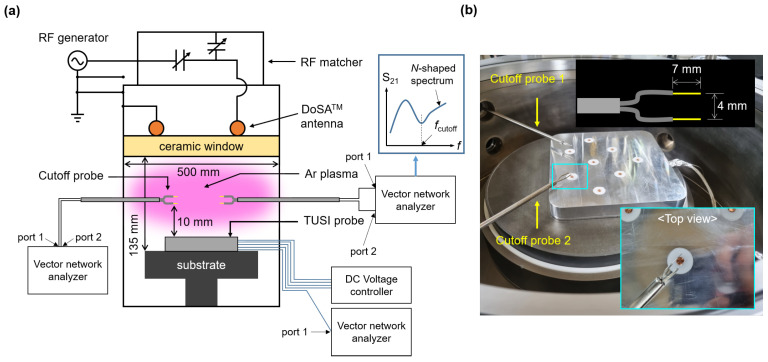
(**a**) Schematic diagram and (**b**) photograph of the experiment setup for the demonstration of the TUSI probe with the cutoff probe.

An inductively coupled plasma source was adopted to demonstrate the TUSI probe. Figure 3a shows a schematic diagram of the experiment setup. The ICP source has a two-turn antenna (DoSA${}^{\mathrm{TM}}$, Plasmart Inc., Daejeon, Republic of Korea) [30] and an RF Matcher (Path Finder, Plasmart Inc., Daejeon, Republic of Korea). The chamber diameter was 500 mm and the substrate the TUSI probe puts on has a distance from the ceramic window of 135 mm. Argon gas of 23 standard cubic centimeter per minutes (sccm) was injected into the chamber via a mass flow controller (STEC SEC, Horiba Ltd., Kyoto, Japan), and an oil rotary pump (W2V40, WSA Co., Ltd., Daejeon, Republic of Korea) drew the gas inside the chamber, and then, the chamber pressure was maintained at 100 mTorr measured by a vacuum gauge (1 Torr Baratron${}^{\mathrm{TM}}$, MKS Inc., Andover, MA, USA). An RF generator with 13.56 MHz (YSR-06MF, YOUNGSIN-RF Co., Ltd., Gyeonggi-do, Republic of Korea) applies the RF power to the DoSA antenna, and then, argon plasma is ignited and sustained.

In this experiment, the cutoff probe is used as the reference probe. Various studies have proved that the cutoff probe is a precise instrument for electron density measurement [31,32,33], so they were secured as reference probes in this demonstration. Since the principle of the cutoff probe is well-described elsewhere [34,35], the following provides a brief explanation. The cutoff probe has radiation and detection antennae connected to ports 1 and 2 of the vector network analyzer. The vector network analyzer sweeps the microwave frequency and records the transmission microwave frequency spectrum (S${}_{21}$) over the frequency. In the S${}_{21}$ spectrum, the measured S${}_{21}$ shows *N*-shaped spectrum having maximum and minimum peaks, and the frequency at the minimum peak is the cutoff frequency ($f_{\mathrm{cutoff}}$) as shown in Figure 3a. At low pressure, the $f_{\mathrm{cutoff}}$ is [34]

(3) $$f_{\mathrm{cutoff}}=f_{\mathrm{pe}}.$$

One can estimate the electron density by using Equations (2) and (3).

Two cutoff probes were installed to minimize plasma perturbation. The cutoff probes 1 and 2 were located above the antennae 3 and 4, respectively, with a distance of 10 mm shown in Figure 3a. Thus, the cutoff probe simultaneously measures the same plasma with the antenna 3 and 4. Each cutoff probe has the same tip geometry; the tip length and the tip distance are 7 mm and 4 mm, respectively. Figure 3b shows the photograph of the cutoff probes and the TUSI probe.

### 3.2. Results and Discussion

**Figure 4:**
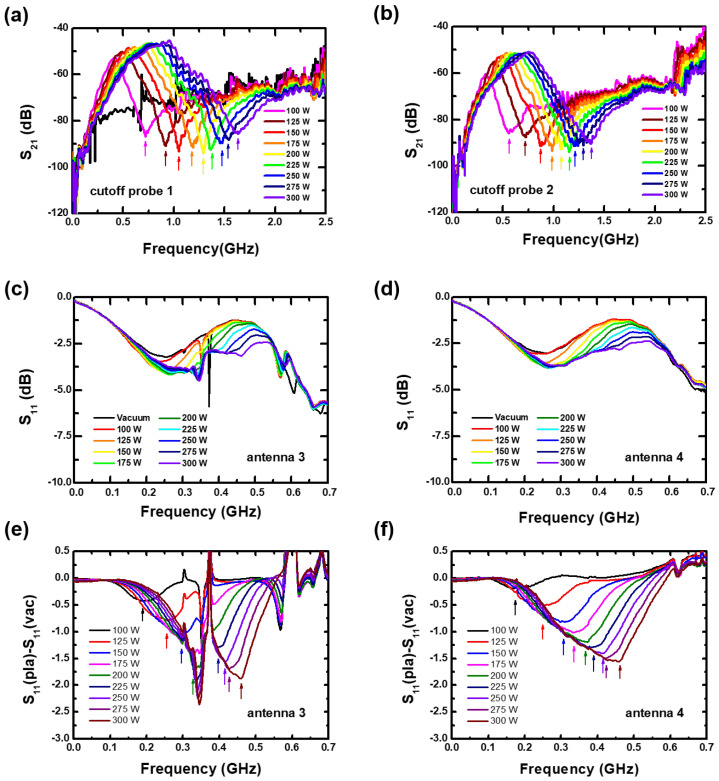
S${}_{21}$ spectra of (**a**) the cutoff probe 1 and (**b**) cutoff probe 2. S${}_{11}$ spectra of (**c**) the antenna 4 and (**d**) the antenna 3. S${}_{11}$(pla)−S${}_{11}$(vac) spectra of (**e**) the antenna 4 and (**f**) the antenna 3. All data are gathered at a pressure of 100 mTorr and various RF power from 100 to 300 W.

Figure 4a,b show the S${}_{21}$ of two cutoff probes with increasing RF power. Clear *N*-shaped spectra emerge in these figures. Here, the marked arrows indicate the $f_{\mathrm{cutoff}}$s at each RF power condition. The $f_{\mathrm{cutoff}}$ shifts toward a high frequency with increasing the RF power and it means an increase in the electron density (Equations (2) and (3)).

Figure 4c,d represent S${}_{11}$ spectra of antenna 3 and antenna 4, which were simultaneously measured with the cutoff probes. Both spectra show evident changes, depending on the RF power compared with the vacuum spectrum, but determining the dip is unclear. It might result from the perturbation by the cutoff probes above each antenna, since a clear peak was observed when the cutoff probes are removed, in the next section. In [12], converted S${}_{11}$ spectrum was introduced [12] for clear dip determination, which is defined as the difference between S${}_{11}$ spectra with plasma and at vacuum conditions, that is, S${}_{11}$(pla)−S${}_{11}$(vac). Here, we used this converted S${}_{11}$ method. Figure 4e,f show the converted S${}_{11}$ spectra where clear peaks marked as arrows emerge. As proved in [12], this peak results from the surface wave resonance and the frequency corresponds to the $f_{\mathrm{SWR}}$.

**Figure 5:**
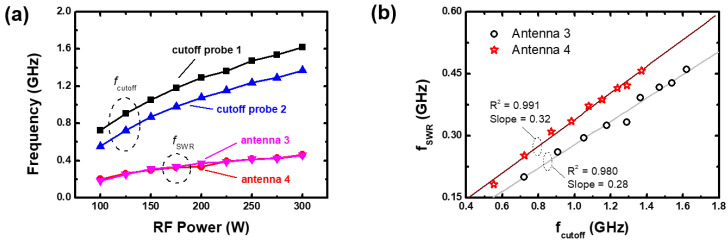
(**a**) Measured cutoff frequencies ($f_{\mathrm{cutoff}}$) and surface wave resonance frequencies ($f_{\mathrm{SWR}}$) with various RF power from 100 to 300 W at pressure of 100 mTorr. (**b**) The $f_{\mathrm{SWR}}$ plot as a function of the $f_{\mathrm{cutoff}}$.

Figure 5a shows the measured $f_{\mathrm{cutoff}}$ and $f_{\mathrm{SWR}}$ at each probe (antenna) with various RF powers as in Figure 4. As the RF power increases, the $f_{\mathrm{cutoff}}$ monotonically increases and $f_{\mathrm{SWR}}$ well follows the trend. There is however the quantitative difference between the antenna 3 and 4, compared it with the difference between the cutoff probes 1 and 2. The cutoff probe 1 measures the $f_{\mathrm{cutoff}}$ as 20% higher than the cutoff probe 2. This indicates that the electron density near the cutoff probe 1 is 45% higher than that near the cutoff probe 2, whereas the densities estimated from antenna 3 and 4 show the slight difference less than a few percent. Figure 5a seems to imply that the TUSI probe requires a correction factor for precise electron density measurement. However, we found that this slight difference might result from the perturbation by the cutoff probe insertion since there is evident difference between the antenna 3 and 4 when the cutoff probes are removed, as shown in the next section. In this study, the demonstration of the TUSI probe unavoidably involved the insertion of the cutoff probes to measure the electron density at a given location. The exact measurement accuracy of the TUSI probe is left as a topic for future research.

Nevertheless, the TUSI probe can serve as a monitoring tool capable of tracking changes in electron density, rather than providing exact measurements of absolute electron density values. To prove that, between them, Figure 5b represents the $f_{\mathrm{SWR}}$ as a function of the $f_{\mathrm{cutoff}}$ as in Figure 5a. It is noted that the $f_{\mathrm{SWR}}$ from antenna 3 and 4 show high linearity designated as the R-square values in this figure, which means that each $f_{\mathrm{SWR}}$ from antennae 3 and 4 play a role as a sensitive indicator of the electron density change. Assuming that all antennae of the TUSI probe have the same accuracy, which is based on the consistent design and characteristics of all antennae, the TUSI probe is able to monitor the electron densities on each antennae, since $f_{\mathrm{SWR}}$ serves as an indicator of variations in electron density and its uniformity.

## 4. Demonstration of the TUSI Probe Working Beneath a Quartz and a SiO${}_{2}$(100 nm)/Si Wafer

### 4.1. Experiment Setup

**Figure 6:**
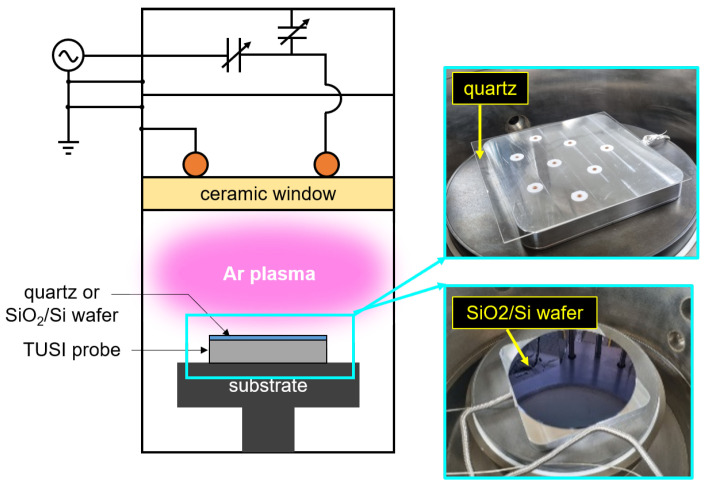
Schematic diagram and photographs of the experiment setup for the demonstration of the TUSI probe beneath a quartz and a SiO${}_{2}$(100 nm)/Si wafer.

In the previous section, the TUSI probe was proved as a monitoring tool for measuring the electron density uniformity. This section focuses on the demonstration of the TUSI probe as a non-invasive in-situ monitoring tool. For realistic demonstration, we put either a quartz or a SiO${}_{2}$(100 nm)/Si wafer on the TUSI probe. The quartz and wafer were used as the substrate in the display and semiconductor fabrication process, respectively. Figure 6 shows a schematic diagram of the experiment setup. The quartz with thickness of 1.0 mm and SiO${}_{2}$(100 nm)/Si wafer of 0.7 mm, which are common specification in fabrications, were used and their area was enough to cover the TUSI probe’s antennae.

### 4.2. Results and Discussion

**Figure 7:**
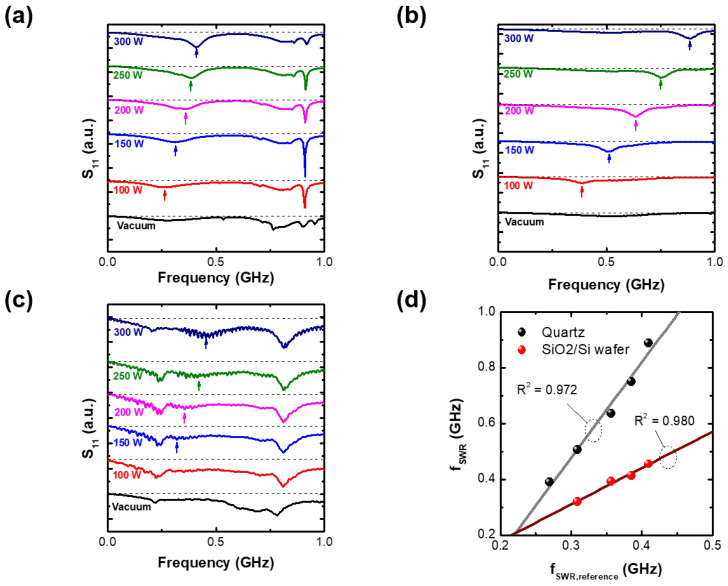
S${}_{11}$ spectra with various RF powers at pressure of 100 mTorr at the RF2 in the cases of (**a**) reference, (**b**) quartz, and (**c**) SiO${}_{2}$(100 nm)/Si wafer, and (**d**) surface wave resonance frequencies ($f_{\mathrm{SWR}}$s) for two cases over surface wave resonance frequency ($f_{\mathrm{SWR},\mathrm{reference}}$) for reference case).

Figure 7a–c show the S${}_{11}$ spectra with various RF powers in the reference, quartz, and Si/SiO${}_{2}$ wafer cases. In the reference case, the S${}_{11}$ spectra show clear $f_{\mathrm{SWR}}$ dip compared with the cutoff probe insertion case (Figure 4c,d). The $f_{\mathrm{SWR}}$ shifts to a high frequency with increasing RF power.

As shown in Figure 7b, a clear $f_{\mathrm{SWR}}$ dip emerges when the TUSI probe is covered by quartz, indicating that the probe operates beneath the quartz during the display fabrication process. Additionally, the $f_{\mathrm{SWR}}$ is higher frequency than that in the reference case at the same RF powers, as shown in Figure 7b. The higher frequency shift in the quartz case results from the sheath effect. The sheath is regarded as the dielectric in microwave range. A previous simulation study [12], where electron density ($n_{e}$) is the input parameter, found the discrepancy between the ideal $f_{\mathrm{SWR}}$ calculated by the input $n_{e}$ as in Equation (1) and the measured $f_{\mathrm{SWR}}$ in the $S_{11}$ spectrum due to a finite boundary effect. Specifically, the discrepancy depended on the sheath width; thinner sheath increases the discrepancy and specifically, lowers the $f_{\mathrm{SWR}}$ from the ideal $f_{\mathrm{SWR}}$. In terms of that, the quartz (dielectric) plays role as the additional sheath and the increase of the sheath width. It makes the $f_{\mathrm{SWR}}$ close to the ideal $f_{\mathrm{SWR}}$, which corresponds to the higher frequency shift of $f_{\mathrm{SWR}}$ in the quartz case compared with the $f_{\mathrm{SWR}}$ in the reference case.

In the case of the Si/SiO${}_{2}$ wafer, noisy fluctuations and ambiguous dips emerges in S${}_{11}$ spectrum as shown in Figure 7c. Despite the smaller dip compared with that in the reference case, the $f_{\mathrm{SWR}}$ is distinguished through magnification and is marked with arrows. The $f_{\mathrm{SWR}}$ increases with the RF power. Comparing the Si wafer case with the reference and quartz cases, it is observed that the $f_{\mathrm{SWR}}$ peak exhibits a low dip for all RF powers. This indicates that the current version of the TUSI probe may not be an effective tool for monitoring semiconductor processes, and suggests that further improvements in sensitivity are required. A lowered dip would result from the skin effect of microwave inside the wafer as it has a small conductivity; a dissipated microwave signal is able to reduce measurement sensitivity. Furthermore, fluctuations in S${}_{11}$ shown in Figure 7c has yet to be understood, but we suppose that they result from a small conductivity of the wafer, compared the vacuum spectra (no plasma in this figure) in the wafer with other cases; the fluctuations emerges only in the wafer case as shown in Figure 7a–c.

To evaluate the linearity of $f_{\mathrm{SWR}}$, we compared the $f_{\mathrm{SWR}}$ values from the quartz and wafer cases to the $f_{\mathrm{SWR}}$ value from the reference case in Figure 7d. The quartz and wafer cases exhibit good linearity, with R-squared values of 0.972 and 0.980, respectively. The differences in $f_{\mathrm{SWR}}$ between these cases and the reference case suggest that the TUSI probe for in-situ use requires a calibration factor for accurate measurement of electron density. However, it is noted that the results demonstrate the TUSI probe’s ability to monitor electron density uniformity beneath a quartz. In the case of a Si/SiO${}_{2}$ wafer, its sensitivity is needed to be improved further.

**Figure 8:**
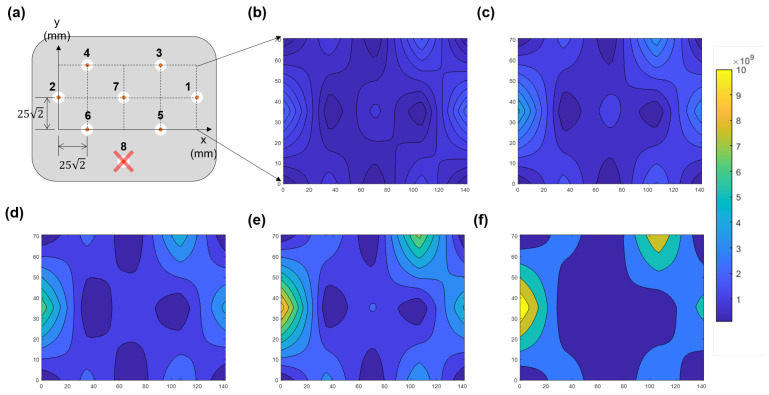
(**a**) Two-dimensional Cartesian coordinate and the antenna positions and numbers of the TUSI probe; the grid distance is 25$\sqrt{2}$ mm and inter-diagonal distance is 50 mm. Measured electron density distribution at the RF power of (**b**) 100 W, (**c**) 150 W, (**d**) 200 W, (**e**) 250 W, and (**f**) 300 W with the same z-scale from 10${}^{8}$ cm${}^{-3}$ to 2 × 10${}^{10}$ cm${}^{-3}$. For clear visualization, the 2-dimensional linear-interpolation method with 50 intervals are adopted in (**b**–**f**). The RF8 malfunctions, so it is excluded.

Figure 8 shows the two-dimensional density map through data processing with increasing RF power in the case of the quartz. The density variation with RF power is well observed in this figure. Hence, it is noted that the TUSI probe is the in-situ non-invasive probe for monitoring the electron density uniformity.

## 5. Conclusions

In this study, we developed the TUSI probe for non-invasive in-situ monitoring of electron density uniformity in plasma processing. By measuring the $f_{\mathrm{SWR}}$ in the S${}_{11}$ spectrum, each antenna of the TUSI probe is able to monitor electron densities and, thereby, electron density uniformity based on the assumption that all antennae have the same accuracy. We validated the TUSI probe through comparison with a precise microwave probe and demonstrated its working beneath a quartz, which is in display fabrication processes. However, we found that the current version of the TUSI probe is not an effective tool for monitoring semiconductor processes and further improvements in sensitivity are required.

## Figures and Tables

**Table 1:** The control logic of a RF Switch. Here, the symbols, ⋆ and -, mean the connection and the isolation, respectively.

V${}_{1}$ (V)	V${}_{2}$ (V)	V${}_{3}$ (V)	RF1	RF2	RF3	RF4	RF5	RF6	RF7	RF8
0	0	0	⋆	-	-	-	-	-	-	-
0	0	1.8	-	⋆	-	-	-	-	-	-
0	1.8	0	-	-	⋆	-	-	-	-	-
0	1.8	1.8	-	-	-	⋆	-	-	-	-
1.8	0	0	-	-	-	-	⋆	-	-	-
1.8	0	1.8	-	-	-	-	-	⋆	-	-
1.8	1.8	0	-	-	-	-	-	-	⋆	-
1.8	1.8	1.8	-	-	-	-	-	-	-	⋆

## Data Availability

The data presented in this study are available on request from the corresponding author.
